# The Artificial Pancreas and Type 1 Diabetes

**DOI:** 10.1210/clinem/dgad068

**Published:** 2023-02-03

**Authors:** Munachiso Nwokolo, Roman Hovorka

**Affiliations:** Wellcome Trust-MRC Institute of Metabolic Science, Box 289, Addenbrooke's Hospital, Cambridge CB2 0QQ, UK; Wellcome Trust-MRC Institute of Metabolic Science, Box 289, Addenbrooke's Hospital, Cambridge CB2 0QQ, UK

**Keywords:** type 1 diabetes, artificial pancreas, automated insulin delivery, hybrid closed-loop

## Abstract

Diabetes technologies represent a paradigm shift in type 1 diabetes care. Continuous subcutaneous insulin infusion (CSII) pumps and continuous glucose monitors (CGM) improve glycated hemoglobin (HbA1c) levels, enhance time in optimal glycemic range, limit severe hypoglycemia, and reduce diabetes distress. The artificial pancreas or closed-loop system connects these devices via a control algorithm programmed to maintain target glucose, partially relieving the person living with diabetes of this constant responsibility. Automating insulin delivery reduces the input required from those wearing the device, leading to better physiological and psychosocial outcomes. Hybrid closed-loop therapy systems, requiring user-initiated prandial insulin doses, are the most advanced closed-loop systems commercially available. Fully closed-loop systems, requiring no user-initiated insulin boluses, and dual hormone systems have been shown to be safe and efficacious in the research setting. Clinical adoption of closed-loop therapy remains in early stages despite recent technological advances. People living with diabetes, health care professionals, and regulatory agencies continue to navigate the complex path to equitable access. We review the available devices, evidence, clinical implications, and barriers regarding these innovatory technologies.

Autoimmune destruction of pancreatic beta cells leaves people living with type 1 diabetes dependent on insulin replacement for life (1). The use of conventional insulin pump therapy and continuous glucose monitoring improves glycemic outcomes and diabetes-related psychosocial outcomes compared with traditional multiple daily insulin injections and capillary glucose monitoring (2-4). Linking pump therapy to continuous glucose monitoring enabled the development of low-glucose suspend (automated insulin infusion suspension at a set low glucose threshold) and predictive low-glucose suspend pump therapy (insulin infusion suspension in anticipation of an algorithm-predicted low and restart once glucose in target) (5, 6). These early automated systems reduced the risk of hypoglycemia but did not address the issue of hyperglycemia (7).

Closed-loop systems are more advanced and mimic endogenous insulin release via automated glucose-responsive insulin delivery (8, 9). Consisting of an insulin pump, a continuous glucose monitor (CGM), and a control algorithm (Fig. 1), they are programmed to minimize both high and low glucose concentrations and achieve improved glycemic control (Fig. 2). The often-used term *hybrid closed-loop* reflects the combination of algorithm-directed insulin delivery and user-initiated prandial bolus doses, the latter required for optimal post-meal glucose control. Fully closed-loop devices requiring no user input and dual hormone closed-loop systems administering insulin plus an adjunctive hormone such as glucagon or pramlintide are under investigation (13-15). The present review is based on a literature search of the Medline and Embase databases using search terms “*artificial pancreas*,” “*automated insulin delivery*,” and “*closed-loop*.” We focused on randomized controlled trials (RCTs). Further reviews are available elsewhere (8, 16, 17).

**Figure 1.**
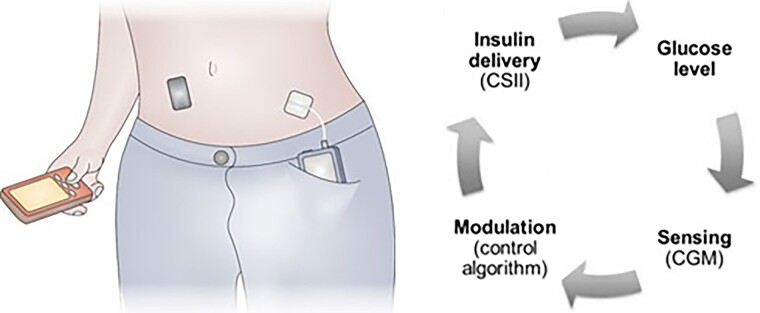
Closed-loop automated insulin delivery system. The continuous glucose monitor (CGM), depicted as the black rectangle, measures interstitial glucose concentrations every 5-10 minutes, and transmits these data to a controller (red handheld device) wirelessly. The controller houses an algorithm engineered to modulate insulin pump delivery (blue device in pocket) based on CGM data. This alters the glucose with the aim of maintaining a set target. The cycle is repeated, hence the term “closed-loop”. Adapted from Hovorka R. Closed-loop insulin delivery: from bench to clinical practice. Nature Reviews Endocrinology, Springer Nature. 2011; 7(7):385-395 (10) reprinted with permission and Bally, L. et al Closed-loop for type 1 diabetes—an introduction and appraisal for the generalist. BMC Med 15, 14 (2017) (11): Open Access CC-BY 4.0. http://creativecommons.org/licenses/by/4.0/.

**Figure 2.**
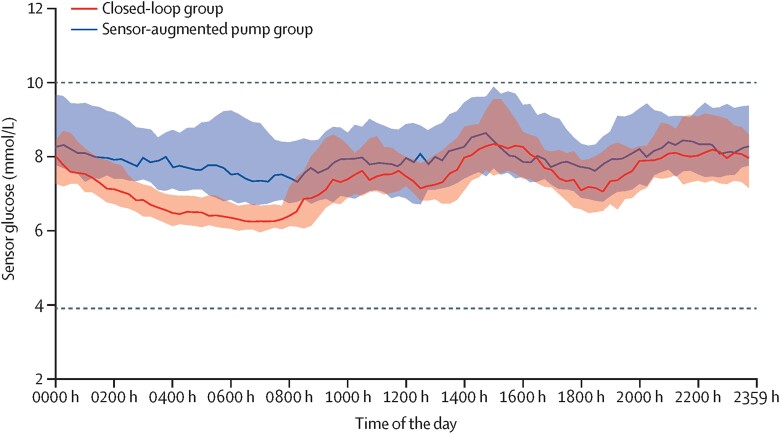
24 hours of closed-loop system versus sensor-augmented pump therapy in adults with type 1 diabetes. Median sensor glucose concentrations (solid red line) and interquartile ranges (red-brown shaded area) during closed-loop insulin delivery and sensor-augmented pump therapy (dark blue line and blue shaded area). Target glucose range between 3·9 and 10 mmol/L (70-180 mg/dL) (dashed lines). Reprinted from The Lancet Healthy Longevity, Vol. 3(3):e135-e142, Boughton. C et al Hybrid closed-loop glucose control compared with sensor augmented pump therapy in older adults with type 1 diabetes: an open-label multicentre, multinational, randomised, crossover study. Lancet Healthy Longevity. Copyright 2022 Mar; 3(3):e135-e142 (12) with permission from Elsevier.

## Closed-Loop Algorithms

Currently available closed-loop technologies use 3 main classes of control algorithm. Model predictive control (MPC) applies a mathematical model of the glucoregulatory system to determine the optimal insulin infusion rate. MPC uses inputs such as glucose measurement and insulin delivery to update model parameters including insulin sensitivity (9). The proportional-integral-derivative (PID) controller calculates insulin delivery based on excursions from target glucose (proportional component), difference between measured and target glucose (integral component), and the rate of glucose change (derivative component) (9). Fuzzy logic algorithms approximate the decision-making of diabetes clinicians (9).

## Available Closed-Loop Systems

**Figure 3.**
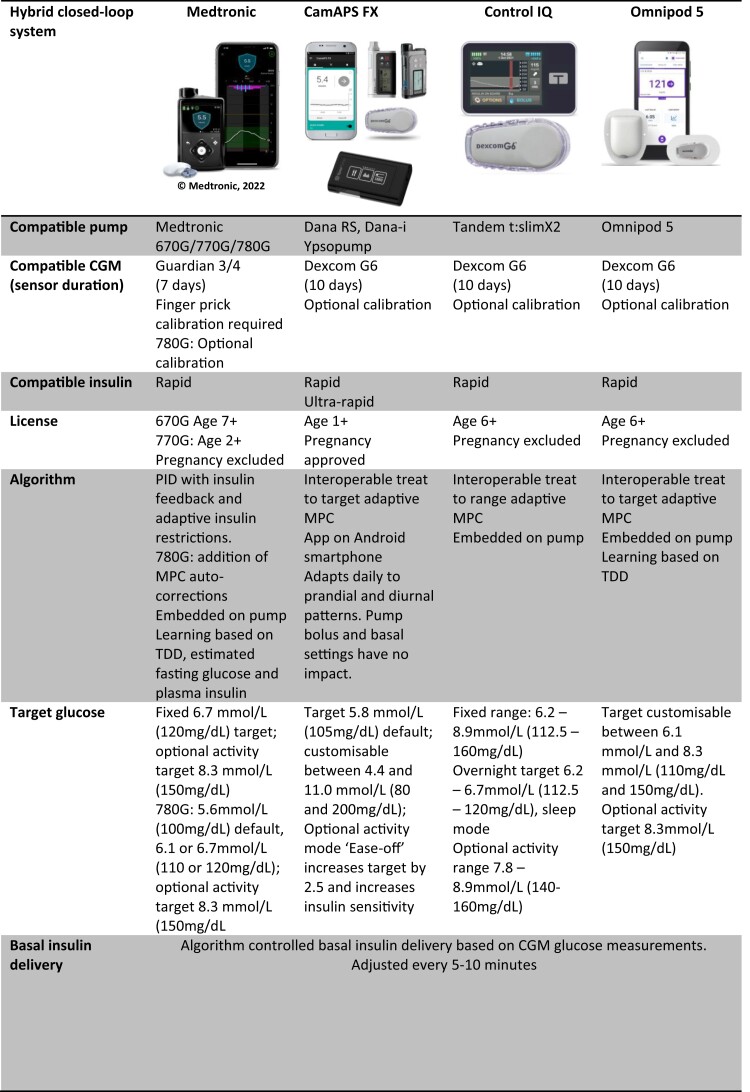

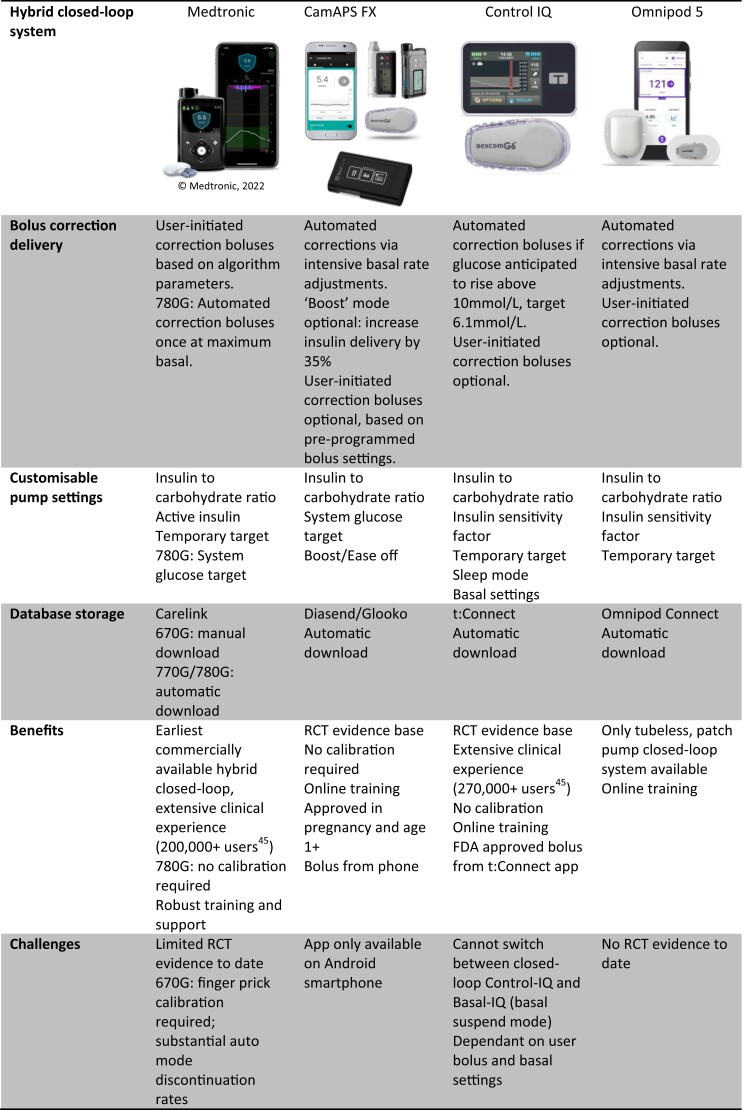
Commercially available hybrid closed-loop AID systems. Medtronic 780 g Advanced Hybrid Closed Loop reproduced with permission of Medtronic Inc.; CamAPS FX app with Dexcom G6 sensor (Dexcom, San Diego, CA, USA), Dana Diabecare RS and Dana-i insulin pumps (Sooil Development Co, Seoul, Korea) and Ypsopump (Ypsomed AG, Burgdorf, Switzerland), reproduced with permission of CamDiab Ltd, Dexcom Ltd, Sooil Development Co and Ypsomed AG; Tandem t:slim X2 insulin pump (Tandem Diabetes Care, San Diego, CA, USA) with Dexcom G6 Sensor (Dexcom, San Diego, CA, USA), reproduced with permission of Tandem Diabetes Care and Dexcom Ltd; Omnipod 5 Automated Insulin Delivery System (Insulet Corporation, Acton, MA, USA) reproduced with permission of Insulet Corporation. AID: automated insulin delivery, CGM: continuous glucose monitor, MPC: model predictive control, PID: proportional-integral-derivative, RCT: randomised controlled trial, TDD: total daily dose (insulin).

The Medtronic 670G (Minimed Medtronic, Northridge California) was the first commercially available hybrid closed-loop system (18). Cleared by the U.S. Food and Drug Administration (FDA) and Conformitè Européenne (CE) marked for ages 7 and above, the 670G was followed by the 770G (FDA approved, licensed for age 2 and above) and 780G (CE marked, licensed for age 7-80 years) (Fig. 3). Medtronic's PID algorithm is embedded within the pump. Initial set-up is based on total daily insulin dose and an estimate of fasting glucose and plasma insulin concentration. The compatible Guardian 3 CGM, which lasts 7 days and requires at least 2 calibrations per day, has been recently upgraded to Guardian 4, requiring no calibrations. McAuley et al conducted a 6-month RCT of the 670G hybrid closed-loop system versus conventional pump or multiple daily injection (MDI) therapy in adults (19). The 670G increased time in range (3.9-10 mmol/L; 70-180 mg/dL) of masked CGM during the final 3 weeks of the study (+15%, *P* < .0001), improved glycated hemoglobin (HbA1c) (−0.4%, *P* < .0001) and enhanced diabetes-specific positive well-being (+1.2, *P* < .0048), with no difference in diabetes distress, perceived sleep quality, or cognition (19). In a multicenter RCT of the Medtronic 780 g advanced hybrid closed-loop versus multiple daily injections of insulin plus intermittently scanned glucose monitoring conducted by Choudhary et al, a greater HbA1c reduction was seen with the hybrid closed-loop (−1.42% *P* < .0001) (20).

CamAPS FX (CamDiab, Cambridge, UK) was the first commercially available standalone, app-based MPC algorithm (Fig. 3). Designed to connect to any compatible CGM or insulin pump, the algorithm adapts to daily diurnal and prandial glucose fluctuations. The algorithm is stored on an Android smartphone and is compatible with the Dana RS and Dana I pumps, YpsoPump, and Dexcom G6 CGM (10-day sensor wear and optional calibration). CamAPS FX is CE marked and is the only system licensed in pregnant women and from 1 year upwards (21, 22). CamAPS FX is also the only system where both rapid and ultra-rapid insulin has been cleared for use. In an RCT comparing CamAPS FX hybrid closed-loop and sensor-augmented pump therapy (SAP) in people living with diabetes with an elevated HbA1c (7.5-10%), Tauschmann et al demonstrated that the CamAPS FX hybrid closed-loop system, in adults and children, significantly increased time in range (+10.8%, *P* < 0.0001) and significantly reduced HbA1c (−0.36%, *P* < 0.0001), time below range (−0.83%, *P* < .0001) and time above range (−10.3%, *P* < .0001) when compared to SAP, with no difference in total daily insulin dose (23).

Control IQ (Tandem, San Diego, CA, USA) is an interoperable MPC algorithm, embedded within the Tandem t:slim X2 insulin pump (Fig. 3). The algorithm is programmed with total daily dose and treats to a fixed glucose target range which can be intensified overnight. The system is compatible with the Dexcom G6 CGM. Control IQ is FDA approved, CE marked, and available for use in ages 6 and above, excluding pregnancy. In a 6-month RCT in adults and children comparing Control IQ closed-loop system with SAP therapy, the closed-loop significantly increased time in range (+11%, *P* < .001) (24). Closed-loop reduced time below range (−0.88%, *P* < 0.001), time above range (−10%, *P* < 0.001) and HbA1c (−0.33%, *P* = 0.01) (24). In a 16-week RCT of closed-loop versus SAP in children aged 6 to 13 years, closed-loop increased time in range by 2.6 hours per day (+11%, *P* < .001) (25). Control IQ has also been shown to improve diabetes distress with high scores in trust and usability in older adults (mean age 68 years) (26).

The Insulet Omnipod 5 (Insulet, Billerica, MA, USA) is the first tubeless hybrid closed-loop system and has been recently cleared by the FDA (Fig. 3). The treat-to-target adaptive MPC algorithm is initially programmed with total daily dose and is located on the Omnipod 5 pump and Omnipod 5 application. Omnipod 5 is approved for ages 6 and up, excluding pregnancy. To date there have been no randomized trials with a control group, only a single-arm safety study has been performed (27).

The Diabeloop closed-loop system consists of an MPC algorithm housed on a handset, a compatible Kaleido patch pump or Roche Accu-check pump, and Dexcom G6 CGM (28). In a 3-month RCT in adults, Benhamou et al compared the Diabeloop hybrid closed-loop system to SAP. Diabeloop improved time in range by 9.2% (*P* < .0001) and reduced time below range (glucose < 3.9 mmol/L; < 70 mg/dL) by 2.4% (*P* < .0001) (28).

Other closed-loop systems in early release, development or in the process of applying for regulatory approval include Tidepool Loop MPC algorithm (29), Inreda PID algorithm (30), and the iLet bionic pancreas (Beta Bionics, Concord, USA), which consists of an adaptive MPC algorithm embedded within the iLet device plus a Dexcom G6 CGM. The iLet bionic pancreas requires input of body weight and, in contrast to other hybrid closed-loop systems, allows qualitative meal announcement, whereby the user states the type of meal and whether the carbohydrate content is usual, more, or less for them, replacing quantitative carbohydrate counting. In a 13-week RCT with children and adults comparing the iLet bionic pancreas (insulin only) to usual care (including hybrid closed-loop, SAP, pump without automation, and MDI with CGM), the bionic pancreas reduced HbA1c by 0.5% (*P* < .001), increased time in range by 11% (*P* < .001) with no difference in time below range between groups (31).

Do-It-Yourself Artificial Pancreas Systems (DIY APS) engineered and built by people living with diabetes combine available pump and CGM technology with open-source algorithms held on a smart device. Systems include DIY Loop, OpenAPS, and Android APS (32). In a multicenter RCT comparing open-source automated insulin delivery (OS-AID) to sensor-augmented pump therapy, Burnside et al demonstrated OS-AID significantly increased time in range (+14% *P* < .001) (33).

## Clinical Evidence

Randomized controlled trials (RCTs) and meta-analyses have demonstrated the efficacy and safety of closed-loop systems. Real-world observational data and single-arm studies have also added to the evidence base (18, 27, 34); however, without a comparator control group, efficacy cannot be assessed. RCTs have shown that closed-loop systems significantly lower mean sensor glucose, increase time spent in target range (3.9-10 mmol/L; 70-180 mg/dL) and reduce HbA1c in comparison with multiple daily injections (MDI) or sensor-augmented pump therapy (SAP) (Table 1). Participants with the highest HbA1c and sensor glucose concentrations at baseline have the greatest improvement in proportion of time in target range (28, 41). Differences in study design and baseline characteristics preclude inter-study and device comparisons.

**Table 1.** Meta-analyses of RCTs evaluating safety and efficacy of closed-loop systems

	Meta-analysis features	Closed-loop intervention	Comparator	ΔTime-in-range 3.9-10.0 mmol/L; 70-180 mg/dL	ΔTime-above-range >10.0 mmol/L; >180 mg/dL	ΔTime-below-range <3.9 mmol/L; <70 mg/dL
**Zeng et al 2022 (13)**	17 RCTs*^a^* Follow-up*^b^*: 12 hours - 2 weeks 438 participants Adults and children Primary outcome: TIR High heterogeneity for TIR (DH vs SH *I^2^* = 63%, DH vs CSII/SAP *I^2^* = 83%) except DH vs PLGS *I^2^* = 0%	Dual hormone	Single hormone CL CSII/SAP PLGS	NS + 16.1% + 6.9%	NS −10.2% −6.2%	−1.20% −3.04% −0.81
**Jiao et al 2022 (35)**	11 RCTs Follow-up*^b^*: 8-26 weeks 817 participants Adults and children Primary outcome TIR Low heterogeneity for TIR (*I^2^* = 21%) High heterogeneity for night-time glucose related outcomes (*I^2^* > 50%)	Single hormone	MDI/CSII/SAP/PGLS	+10.3%	−8.9%	−1.09%
**Fang et al 2021 (36)**	12 RCTs Follow-up*^b^*: 12 hours – 8 weeks 344 participants Adults Primary outcome: TIR Moderate heterogeneity for TIR (*I^2^* = 67%)	Single hormone Dual hormone	SAP	+7.9%	—	—
**Eckstein et al 2021 (37)**	6 RCTs of CL during exercise Exercise duration: 40 - 330 min 153 participants Adults and children Primary outcome: TIR during exercise	Single hormone Dual hormone	CSII/SAP/PLGS	+6.2%	—	—
**Pease et al 2020 (38)**	Network meta-analysis 14 RCTs Follow-up*^b^*: 2 – 12 weeks 1043 participants Adults High network heterogeneity for TIR (*I^2^* = 87%)	Single hormone	MDI with SMBG MDI with Flash MDI with CGM CSII with CGM/Flash/SMBG CSII with CGM	+17.9% +13.3% +12.8% +10.6% +8.8%	NS NS NS NS NS	NS NS NS NS NS
**Bekiari et al 2018 (39)**	40 RCTs Follow-up*^b^*: 12 hours – 12 weeks 1027 participants Adults and children Primary outcome TIR High heterogeneity for TIR (*I^2^* = 81%)	Single hormone Dual hormone	MDI/CSII/SAP/LGS	+9.6%	−8.5%	−1.49%
**Weisman et al 2017 (40)**	24 RCTs Follow-up*^b^*: 12 hours – 12 weeks 585 participants Adults and children Primary outcome TIR*^c^* High heterogeneity for TIR*^c^* (*I^2^* = 84%) and TBR (*I^2^* = 94%)	Single hormone Dual hormone	CSII/SAP/LGS	+12.6%*^c^*	—	−2.45%

Closed-loop meta-analyses data including number of RCTs, follow-up period, participants, primary outcome and/or heterogeneity and the resulting increase (+) or decrease (−) in percentage time in range, time above range, or time below range with the closed-loop intervention versus the comparator.

Abbreviations: CSII, continuous subcutaneous insulin infusion therapy; Flash, flash glucose monitoring; LGS, low-glucose suspend therapy; MDI, multiple daily injections therapy; NS, non-significant; PGLS, predictive low-glucose suspend therapy; RCT, randomized controlled trial; SAP, sensor-augmented pump therapy; SMBG, self-monitoring of blood glucose; TIR, time in range (3.9 to 10 mmol/L; 70-180 mg/dL); TBR, time below range (<3.9 mmol/L; <70 mg/dL).

Zeng et al included studies with and without exercise.

Duration of each intervention arm or phase, excluding washout. Closed-loop trials only.

Weisman et al included studies defining TIR as 3.9-10.0 mmol/L (70-180 mg/dL) or 3.9-8.0 mmol/L (70-144 mg/dL).

Several meta-analyses have validated that closed-loop systems have a favorable effect on time in range (significantly higher time spent 3.9-10.0 mmol/L; 70-180 mg/dL), lower time spent in hypoglycemia (< 3.9 mmol/L and < 3.0 mmol/L; < 70 mg/dL and < 54 mg/dL) and hyperglycemia (> 10 mmol/L; > 180 mg/dL), improve HbA1c, and may result in fewer adverse events (13, 36-40) (Table 1). In a recent meta-analysis of 10 RCTs, Jiao et al reported that closed-loop systems increased time in range by 2 hours and 27 minutes per day, reduced glycemic variability (coefficient of variation of glucose reduced by 1.41) and improved HbA1c (−0.30%) with no difference in total daily insulin dose (35).

Dual hormone (DH) systems, combining insulin with other hormones such as glucagon or pramlintide, are in theory closer to mimicking the hormonal responses of the pancreas than single hormone closed-loop (SH) systems; however, the data are conflicting. A recent meta-analysis of 17 dual hormone RCTs, found no significant difference in time in range between single and dual hormone systems (13). Zeng et al included 9 trials comparing both DH and SH. A small reduction in time below range (< 3.9 mmol/L; < 70 mg/dL) was noted in DH compared with SH; however, this was accompanied by a higher risk of gastrointestinal symptoms (13). When stratified by study setting, DH showed a significant improvement in time in range in the inpatient setting compared to SH but not in the outpatient setting (13), reflecting the practical challenges of real-world application. Two earlier meta-analyses by Bekiari et al and Weisman et al reported that DH systems were associated with an increased in time in range in a DH versus SH subgroup analysis; however, these 2 meta-analyses respectively only included 4 and 2 trials that comparatively assessed SH vs DH (39, 40). Exercise-induced glucose variability has been shown to challenge closed-loop systems and potentially lead to hypoglycemia (37). Dual hormone systems have been shown to moderately reduce time below range (13); however, an exercise-focused meta-analysis found no difference between SH and DH. Similar to SH systems, DH closed-loop has been shown to significantly improve glucose control when compared with pump alone or sensor-augmented pump therapy; however, longer trials with more standardized reporting are required to support clinical adoption of DH closed-loop systems and to clarify who will most benefit.

Fully closed-loop systems are the next step toward the completely automated approach, requiring no input from the person wearing the devices (14). Multiple input algorithms are also being explored (42). The addition of inputs such as pulse rate, sweat, movement, and step count to glucose data may address some of the challenges seen with prandial glycemic variability, activity, and other stressors.

## Psychosocial Impact

Positive psychosocial outcomes of closed-loop systems support clinical adoption. Barnard et al found that 20 out of 24 participants would recommend closed-loop systems in a qualitative study examining psychosocial experience (43). Participants felt that closed-loop systems improved blood glucose control, reduced worry, and improved overnight control leading to improved daily functioning and better sleep; however, they also reported technical difficulties, alarm intrusiveness, and cumbersome equipment (43). A systematic review of 19 quantitative and qualitative studies investigating the values and preferences of people living with diabetes, found that optimal glycemic control was prioritized over other significant drivers such as avoidance of complications, glycemic variability, hypoglycemia reduction, and treatment burden (44). Recent non-randomized studies have suggested that when compared to baseline conventional therapy, closed-loop systems improve diabetes-related psychosocial outcomes such as diabetes distress, hypoglycemia confidence, insulin delivery satisfaction, and system usability but no significant changes in sleep parameters (26, 45). However, a meta-analysis of closed-loop versus other treatment modalities including sensor-augmented pump therapy and multiple daily injections found that quality of life, satisfaction with diabetes and diabetes distress assessments were not significantly different (35). Any positive impact on quality of life or reduction in distress may have been counterbalanced by the challenges of understanding and using the advanced technology and device burden.

Healthcare professional attitudes also impact on the interaction and communication with individuals using advanced technologies. Qualitative data by Lawton et al reported assumptions on who would use closed-loop technology effectively were held by healthcare professionals in the research setting, perhaps influencing access (46). A survey evaluating UK healthcare professional views on do-it-yourself (DIY) automated systems reported 97% felt that healthcare professionals should learn more about DIY systems to support those using them (47). Evidence-based yet individualized communication is warranted between people using advanced technologies and healthcare professionals (46, 48).

## Clinical Adoption

The National Diabetes Audit (NDA) of England and Wales reported 9.8% of people living with diabetes achieve the National Institute for Health and Care Excellence (NICE) recommended HbA1c target of ≤ 6.5% (< 48 mmol/mol), while 32.3% achieve ≤ 7.5% (58 mmol/mol) (49). The US T1D Exchange and Prospective Diabetes Follow-up (Austria and Germany) registries reported that 21% and 39% achieved the ADA HbA1c target (< 7% for adults; < 7.5% for children and older adults aged ≥ 65 years) respectively (50). A majority of people living with type 1 diabetes are at significantly higher risk of microvascular and macrovascular complications (51, 52). Hybrid closed-loop systems have set a new benchmark for optimal time in range, an international consensus recommended glucose metric (53) shown to correlate with diabetes complications and HbA1c (54, 55); however, significant challenges with heterogeneous data, clinical implementation, and accessibility remain.

In England and Wales, 2020-2021 NDA data have shown that insulin pumps and CGM are more likely to be used by adults of White ethnicity, the least deprived, and the youngest age groups (49). Significant geographical variation was also recorded, with insulin pump provision ranging from 4% to 30% and intermittently scanned or flash glucose monitoring availability ranging from 13% to 55% across regions. In March 2022, the UK NICE published new guidance recommending NHS-funded flash or CGM for all adults living with type 1 diabetes (56), an important step toward more equitable access. Notably, despite a technology appraisal recommending pump therapy published by NICE in 2008, less than 10% of adults living with type 1 diabetes in England and Wales are on an insulin pump (49). The NDA 2020-2021 data showed that 58 810 adults on multiple daily injections met NICE criteria for an insulin pump (49).

Global variation in commercial availability and affordability impacts device access. In the United States, an estimated 62% of adults living with type 1 diabetes use an insulin pump, compared with 5% to 19% in Europe (57, 58). An analysis of 28 019 US adults and children with type 1 diabetes in the T1D exchange database reported 7% hybrid closed-loop use compared with 44% using pump and CGM without automated delivery and 49% using MDI and CGM. Within the hybrid closed-loop group, inequities in age, ethnicity, education, and insurance status were demonstrated (59). Individuals using hybrid closed-loop were more likely to be under the age of 18 years (58%) and White (8% of the Non-Hispanic White population used hybrid closed-loop compared with 3% of the Non-Hispanic Black population, *P* < .001) (59). In the United States, access to hybrid closed-loop therapy depends on insurance status while in other countries, including the United Kingdom and Denmark, public funding is available under specific criteria; however, regional budgets and policies may override clinical recommendations contributing further to geographical inequities (58-60). Further barriers to uptake and continuation include concerns around wearing the devices and related stigma (61). Technical challenges, such as significantly low total daily insulin dose requirements, as seen in small children, can impact closed-loop accuracy (62). Attention should be paid to the lowest basal rate and lowest basal and bolus increment when selecting a device (62).

Equitable access and uptake rely on effective healthcare professional training and health service infrastructure to deliver and support use of advanced diabetes devices (48, 63). Complex cases should be discussed in a multidisciplinary format with access to advanced technology specialists (48). Optimal care for those using advanced technologies also comprises standard diabetes care processes, including blood pressure, renal, and retinopathy screening and foot risk stratification alongside well-being and technical support (17, 48, 64).

Training support and educational documents are available for healthcare professionals and users ranging from commercialized online training, for example, CamDiab certified modules, Medtronic Clinical Education, Tandem Diabetes Care, Omnipod virtual training hub, Libre Academy to specialized support and guidance, for example, Diabetes Technology Network (DTN-UK; an organization designed to educate and support health care professionals involved in technology delivery) and the CARES paradigm, a clinical guide to understanding and comparing closed-loop systems (64).

## Conclusions

Closed-loop systems continue to evolve and promote improved glycemic and psychosocial outcomes. The heterogeneous data tend not to include diverse ethnic and socioeconomic populations, limiting the generalizability of study findings. Technological advances, expanding interoperability and more standardized, reflective data will facilitate individualized care offered with multidisciplinary support. Closed-loop technologies may offer meaningful respite for people living with diabetes aiming to maintain the glucose targets required to avoid long-term complications. Providing equitable yet cost-effective access to those that would benefit the most remains a challenge.

## Data Availability

Data sharing is not applicable to this article as no datasets were generated or analyzed during the current study.

## Abbreviations

CE mark: Conformitè Européenne mark
CGM: continuous glucose monitor
DH: dual hormone
DIY: do-it-yourself
FDA: U.S. Food and Drug Administration
HbA1c: glycated hemoglobin
MDI: multiple daily injection
MPC: model predictive control
NDA: National Diabetes Audit
NICE: National Institute of Clinical Excellence
PID: proportional-integral-derivative
SAP: sensor-augmented pump therapy
SH: single hormone
